# 1004 Rise and Shine: A Multidisciplinary Burn Mobility Initiative

**DOI:** 10.1093/jbcr/iraf019.535

**Published:** 2025-04-01

**Authors:** Susan Smith, Rachel Gonzalez, Brooke Bozoian

**Affiliations:** Orlando Regional Medical Center; Orlando Regional Medical Center; Orlando Regional Medical Center

## Abstract

**Introduction:**

Mobilization is defined in many ways however, in the stepdown unit setting, the study adapted the definition to encompass achievement of vertical functional maneuvers aimed at activity occurring out of bed. Burn patients are a heterogenous group when considering mobility due to differing location of burns, wound care needs, pre-injury mobility, pain history, and levels of family support. Barriers to mobility in the critically ill burn population have been described in the literature as inadequate equipment or staff, pain and sedation, timing of dressings, and surgical considerations. Multidisciplinary coordination and collaboration are essential. The burn trauma stepdown has less than 50% compliance with the hospital’s “Rise and Shine” initiative, supporting the need to evaluate and improve mobility in the burn population. The purpose for this exploratory study is to assess multidisciplinary opinions on barriers to mobility on a burn stepdown (intermediate care) unit to provide background for developing a guideline.

**Methods:**

A multidisciplinary survey was created to assess perceived barriers to and compliance with out of bed mobility in the adult burn patient population on the burn trauma stepdown unit of a single verified burn center. Target population included burn trauma stepdown staff registered nurses and occupation and physical therapist and therapy assistants routinely assigned to the burn trauma stepdown.

**Results:**

The survey was released to 32 full time burn registered nurses and burn therapy staff to include 3 physical therapist, 1 physical therapy assistants, 4 occupational therapist and 2 occupational therapy assistants. There were 24 total responses, with a 57% response rate. Most impactful identified barriers to mobilization were pain, sedation from pain medications, timing of dressings and staffing. Respondents indicated that therapists and providers are primarily responsible for educating burn patients on the importance of mobility.

**Conclusions:**

This study appears to be the first to specifically address mobility in the stepdown (intermediate care) adult burn population. Survey results were consistent with current literature describing barriers to mobility in the critically-ill burn population. Pain, surgical considerations and dressing change related issues dominate the responses. Results also illuminated the need to improve multidisciplinary coordination when planning for burn patient mobility. The survey findings were cited in support of decreasing nurse patient ratios and for increasing the number of dedicated burn therapists.

**Applicability of Research to Practice:**

This survey illuminated areas of concern and educational gaps for our burn stepdown nursing and rehab staff. It provides the foundation for a prospective study examining mobility following the roll out of decreased nurse to patient ratios and targeted multidisciplinary burn mobility education.

**Funding for the Study:**

N/A

